# Refining Cancer Management Using Integrated Liquid Biopsy

**DOI:** 10.7150/thno.40677

**Published:** 2020-01-16

**Authors:** Juhui Qiu, Jianxiong Xu, Kang Zhang, Wei Gu, Liming Nie, Guixue Wang, Yang Luo

**Affiliations:** 1 Key Laboratory for Biorheological Science and Technology of Ministry of Education, State and Local Joint Engineering Laboratory for Vascular Implants, Bioengineering College of Chongqing University, Chongqing, 400044, China.; 2 State Key Laboratory of Molecular Vaccinology and Molecular Diagnostics & Center for Molecular Imaging and Translational Medicine, School of Public Health, Xiamen University, Xiamen 361102, China.; 3 Nuclear Medicine and Molecular Imaging Key Laboratory of Sichuan Province, Department of Nuclear Medicine, the Affiliated Hospital, Southwest Medical University, Luzhou, Sichuan, 646000, China.; 4 Center for Laboratory Medicine, Medical College of Chongqing University, Chongqing, 400044, China.

**Keywords:** Combined detection, Cancer management, Circulating tumor DNA, Circulating tumor cell, Extracellular vesicle.

## Abstract

Liquid biopsy has emerged in the last ten years as an appealing noninvasive strategy to support early cancer diagnosis and follow-up interventions. However, conventional liquid biopsy strategies involving specified biomarkers have encountered unexpected inconsistencies stemming from the use of different analytical methodologies. Recent reports have repeatedly demonstrated that integrated detection of multiple liquid biopsy biomarkers can significantly improve diagnostic performance by eliminating the influence of intratumoral heterogeneity. Herein, we review the progress in the field of liquid biopsy and propose a novel integrated liquid biopsy framework consisting of three categories: elementary, intermediate, and advanced integration. We also summarize the merits of the integration strategy and propose a roadmap toward refining cancer diagnosis, metastasis surveillance, and prognostication.

## Introduction

**Figure 1:**
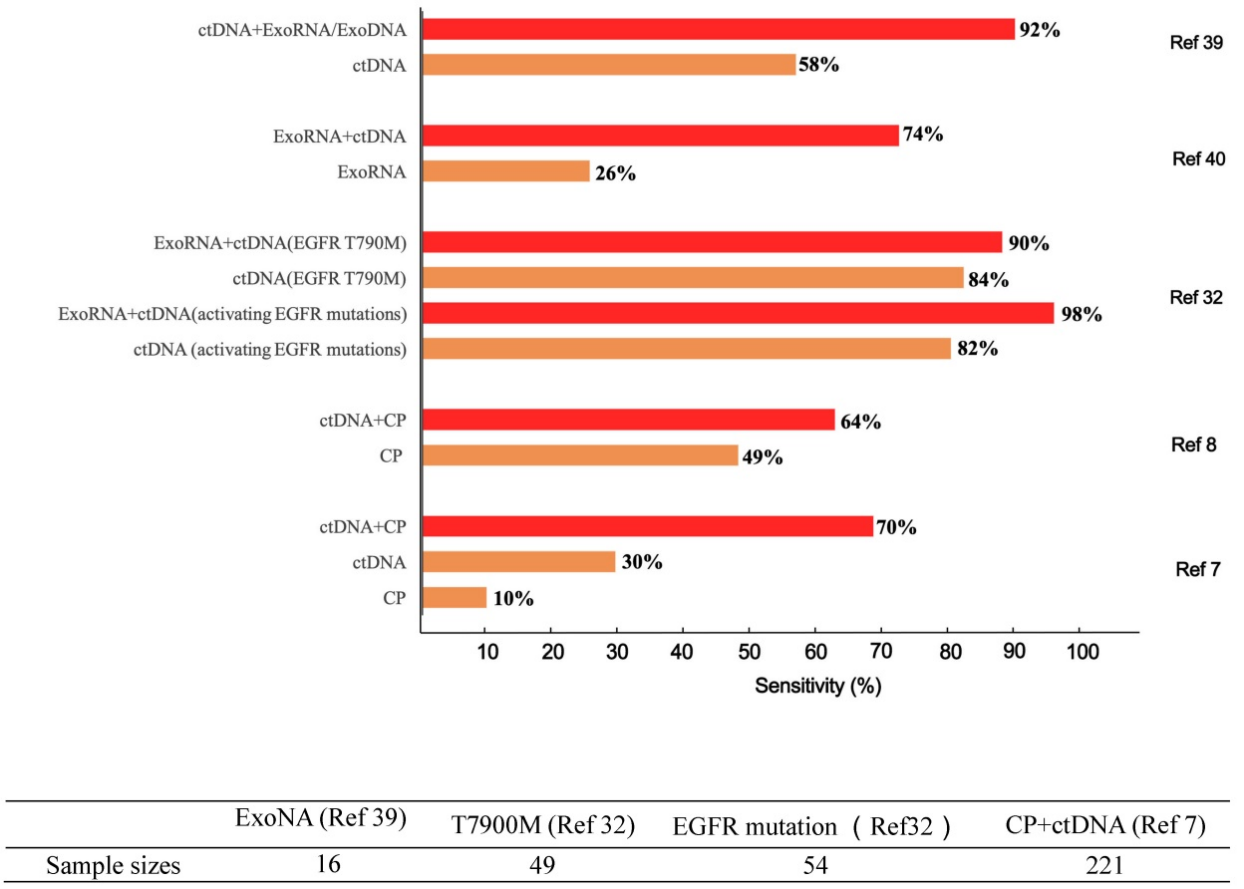
**Differences in diagnostic sensitivity between integrated and single tumor-associated material detection.** The X axis denotes the sensitivity, and the Y axis represents different TAM combination panels. TAM, tumor-associated material. The combination of exosomes and DNA greatly improved cancer detection in 16 sample sizes 39. Compared to single-marker detection in the T7900M locus, the combination of exosomes and ctDNA improved the detection of positive patients from 41 to 44 out of 49. Likewise, for the EGFR activation site, detection was increased from 44 to 53 out of 54 patients 32. Meanwhile, the combined detection of protein and ctDNA also increased the sensitivity from 66 to 141 of 221 patients 7. The sample sizes are as follows.

Bodily fluids, such as blood, urine, cerebrospinal fluid, and saliva, contain numerous biomarkers corresponding to patient-specific pathological information. From the perspective of biodetection, any biomarkers that are highly associated with tumor growth and metastasis, including circulating proteins (CPs), circulating tumor DNA (ctDNA), circulating tumor RNA (ctRNA), extracellular vesicles (EVs), circulating tumor cells (CTCs), and tumor-educated blood platelets (TEPs), can be indicators of carcinomas. Liquid biopsy has been recognized as one of the most promising strategies for conquering cancers owing to its unparalleled advantages over classical solid biopsy in improving patient compliance, partially due to its noninvasive sample collection and reduced potential for surgical complications 1-3. Unfortunately, discordant detection results are frequently encountered when those single biomarkers are analyzed; this inconsistency probably stems from tumor heterogeneity as well as varied analytical methodologies 4. In particular, undesirable inconsistencies from two Clinical Laboratory Improvement Amendments (CLIA)-certified laboratories were observed in the analysis of 40 clinical samples from prostate cancer (PC) patients 5. Only 22% of the mutations were concordant between two commercially available next-generation RNA sequencing platforms^6^. This discordance casts a shadow over the future of liquid biopsy. Several recent publications have revealed that the joint detection of several biomarkers or the use of multiple methods with different principles can significantly improve the sensitivity with which cancers can be differentiated at early stages 7, 8 (Figure 1).

**Figure 2:**
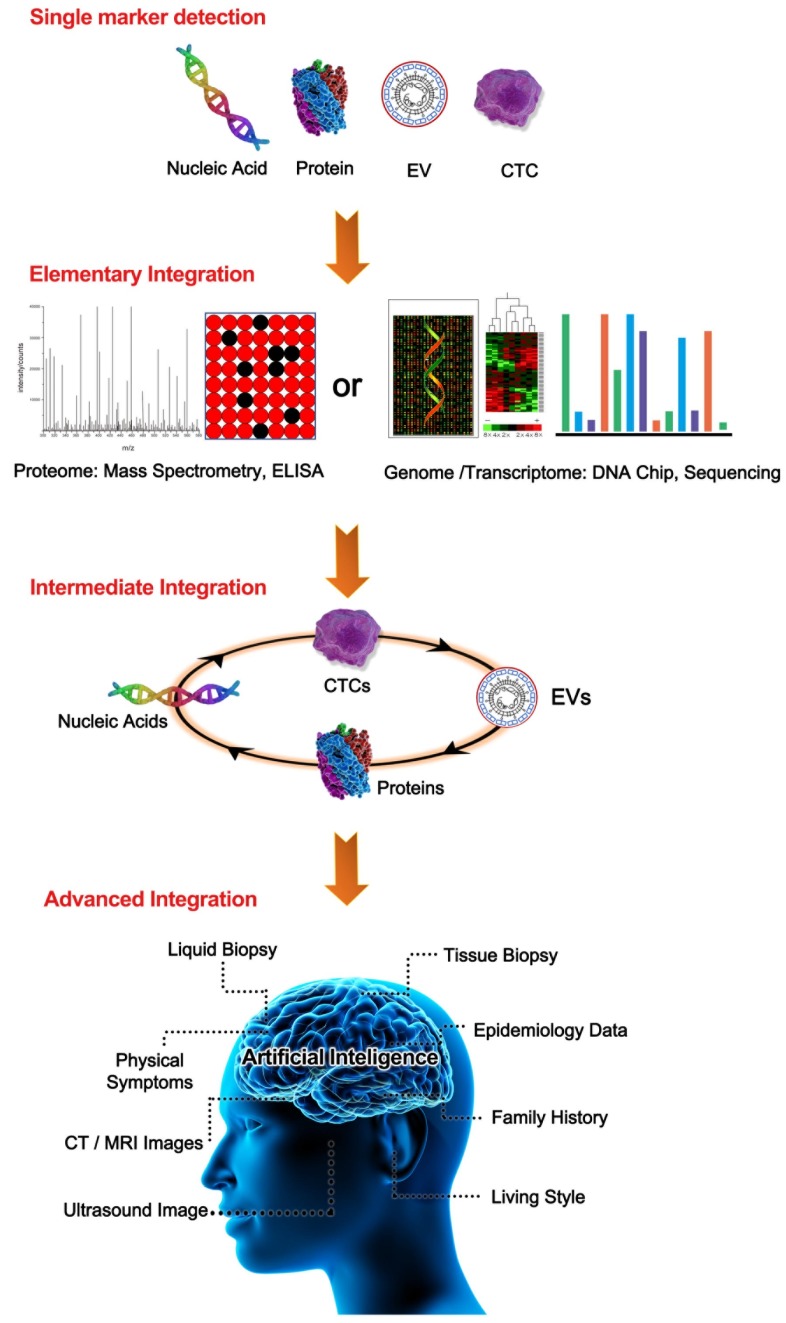
**A summary of the categories of liquid biopsy integration.** From the top to the bottom are single biomarker detection, elementary integration, intermediate integration, and advanced integration. In the single biomarker group, any TAM was used alone, such as a single nucleic acid, CTC, EV, or CP. In the elementary integration group, representative TAM integrations through omics data from either proteomics or nucleic acid sequencing-based genomics were demonstrated. In the intermediate integration group, two/multiple TAMs of nucleic acids, CPs, CTCs, or EVs were detected by combining them in an all-in-one format. In the advanced integration group, intermediate integration could be further enhanced by combination with other available data such as tissue biopsies, life style, and medical imaging with the help of artificial intelligence.

This review will intensively discuss the hurdles facing liquid biopsy prior to its clinical application and propose potential solutions. Here, we propose a novel analytical framework, termed “integrated liquid biopsy”, that could be a useful toolkit bridging conventional liquid biopsy and clinical requirements. “Integrated liquid biopsy” is defined as integrating multiple liquid biopsy biomarkers or detection methods for improved analytical sensitivity and specificity to refine cancer management. Since a single biomarker is inefficient in accurately identifying most cancers, integrated liquid biopsy using multiple markers might be a promising method to facilitate early detection and treatment of cancer. According to the complexity of the combined data types, we categorize integrated liquid biopsy into the following three groups: 1) Elementary integration refers to the combination of biomarkers or methods of the same type,* e.g.*, protein-protein, RNA-RNA or DNA-DNA combinations 9, 10. With the great progress of high-throughput methods for nucleic acids and proteins, such as sequencing and proteomics, implementing elementary integration in the clinic has become a well-established trend in the broader shift toward precision medicine. 2) Intermediate integration denotes the combination of two or more types of liquid biopsy biomarkers or methods, *e.g.*, protein-DNA combinations 8, 11. The role of intermediate integration has been demonstrated over time in the clinical diagnosis of early-stage cancers (Figure 1), but this strategy has yet to be exploited in monitoring cancer progression and therapy. 3) Advanced integration represents the combination of results from liquid biopsy and other methods to map the cancer on the temporal and spatial scales (Figure 2). This strategy was first introduced in coronary artery disease diagnosis, in which context it was used to assess the prevalence and clinical impact of cardiovascular diseases by combining data from DNA sequencing, serum lipoproteins and electronic health records 12, 13. The concept of advanced integration idea was also applied by another research group to determine the outcome probabilities for individual patients through a statistical model based on risk predictors that incorporate ctDNA and medical imaging acquired over time 14.

## Integrated liquid biopsy improves the efficiency of early cancer detection

Liquid biopsies have been regarded as robust tools for routinely screening and identifying tumors before symptoms appear. Historically, the detection of a single CP (*e.g.*, CA19-9, CEA, AFP, and prostate-specific antigen (PSA)) achieved prevalence due to its convenience and low cost 15, 16. However, the limited specificity of such markers has been widely recognized. The discovery of new molecular biomarkers overcomes such drawbacks due to their intimate association with cancer-related genetic alterations. For instance, the use of two or more carcinogenesis-related gene mutations 17, 18 or ctDNA methylation patterns reflecting primary tumor sites has frequently been reported 19, 20. Meanwhile, Glypican-1^+^ exosomes containing diverse RNA and proteins may distinguish healthy subjects from patients with pancreatic cancer 21, 22. Other research has used proteomic analysis to identify specific molecules in the exosome “surfaceome” that are candidate biomarkers for pancreatic ductal adenocarcinoma^23^.

Unfortunately, the enormous heterogeneity of tumors renders single-biomarker-based detection potentially inaccurate; for this reason, combined detection of multiple biomarkers is becoming widespread. A protein panel comprising several CPs was proposed as the prototype of preliminary integration 24. In particular, a prostate health index combining prostate-specific antigen (PSA), p2PSA, and free PSA has been proposed for differentiating PC from benign prostatic hyperplasia 25. The entire spectrum of structural changes to PSA in blood effectively discriminated high-grade PC (Gleason≥7) from low-grade/benign disease (Gleason=6) 26. More recently, profiling of a series of biomarkers from cerebrospinal fluid provided support for tracking and monitoring the evolution of glioma 27. The integration of two exosome-derived proteins (Glypican-1 and CD63) facilitated the diagnosis of pancreatic cancer 28, 29. Furthermore, A similar result showed that integrating the exosomal RNAs (exoRNAs) miR-122 and miR-148a with α-fetoprotein accurately distinguished early hepatocellular carcinoma from liver cirrhosis 30. Additionally, a diagnostic model based on expression levels of ten exosomal miRNAs exhibited astonishing accuracy (sensitivity of 0.99 and specificity of 1.00) in an early-stage ovarian cancer cohort 9.

Regarding intermediate integration, CP-ctDNA integration is particularly attractive because ctDNA indicates specific genetic alterations and because the relatively high concentrations of CPs compensate for the drawback of low ctDNA abundance. Joshua et al. reported that the conjunction of *kirsten rat sarcoma viral oncogene* (*KRAS*) mutations and CA19-9 provided an improved sensitivity of 64%, compared to 30% for *KRAS* alone 8. These results validated the potential for the integrated detection of exoRNA profiles and CPs in identifying the presence or severity of solid tumors and hematological malignancies 31. In a study of non-small cell lung cancer (NSCLC), the diagnostic sensitivity of exoRNA-ctDNA integration for recognizing activating epidermal growth factor receptor (EGFR) mutations surpassed that of ctDNA alone (98% vs 82%) 32. A ctDNA-based blood test employing three prototype sequencing assays (single nucleotide variants/indels, copy number variation, and methylation) also detected 20 tumor types at various stages with high specificity 7. In the advanced category, a multianalyte blood test called CancerSEEK, integrating CPs and ctDNA profiles in conjunction with artificial intelligence (AI), yielded satisfactory sensitivity ranging from 69% to 98% for the detection of five cancer types (ovary, liver, stomach, pancreas, and esophagus) while maintaining a high specificity of 99% (Figure 1) 7.

Despite the popularity of liquid biopsy in a clinical setting, the conventional detection of a single biomarker has encountered numerous obstacles in assessing samples with tissue or organ heterogeneity. These reported discrepancies among different detection approaches primarily originate from the small amounts and easy degradability of these biomarkers, features that severely compromise their value in indicating abnormal clonal cell proliferation. The latest studies have revealed that a CP-ctDNA integration strategy significantly improves the sensitivity of earlier cancer detection without substantially decreasing specificity. Additionally, integrated assays of CPs and genetic alterations further localize the original organs of these cancers, which could greatly benefit further therapy. Meanwhile, the CP-ctDNA integration concept could be expanded to other liquid biomarkers, such as metabolites, mRNA transcripts, miRNAs, methylated DNA sequences, or markers in EVs to increase the efficiency of early cancer detection.

## Liquid biopsy integration promotes the management of cancer therapy

**Figure 3:**
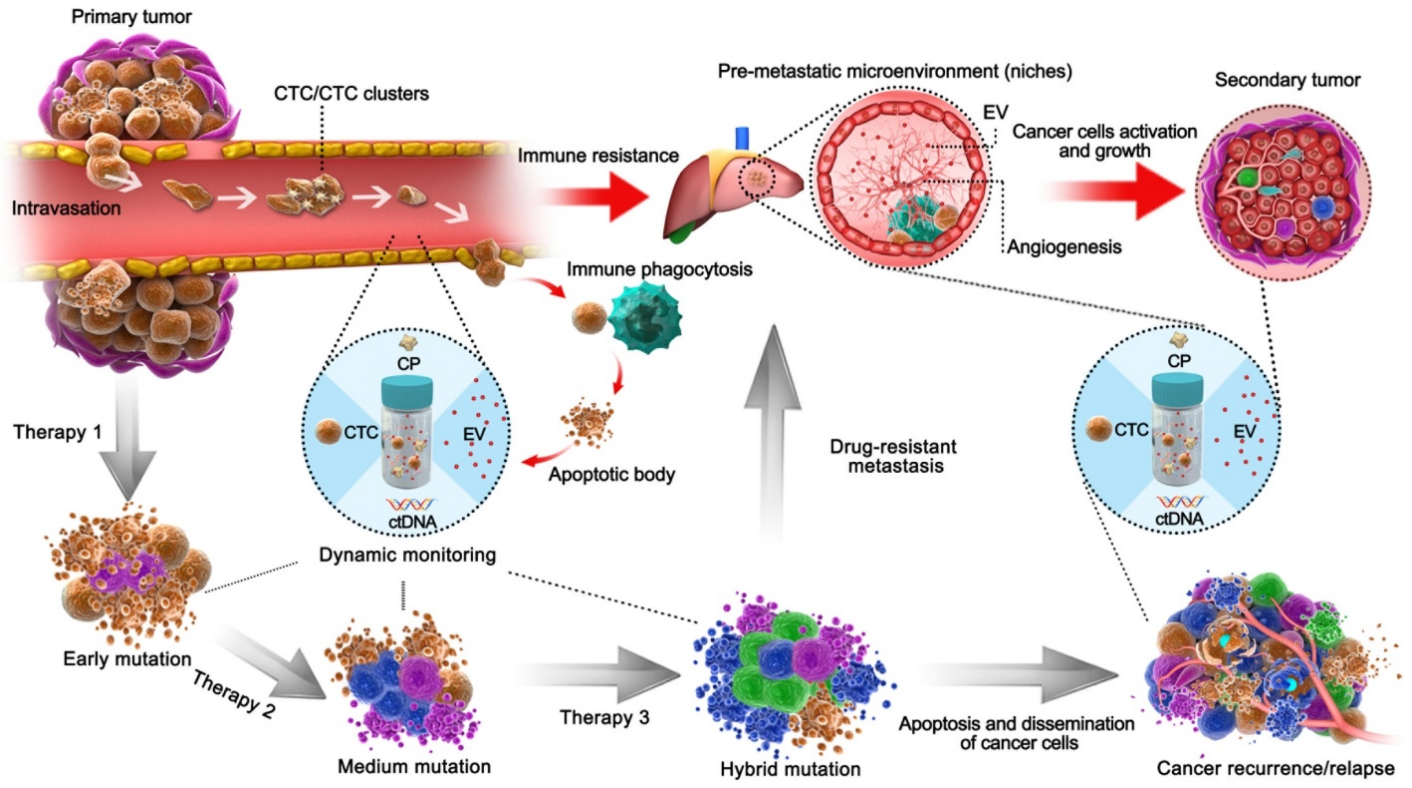
** Schematic liquid biopsy during the multi-step process of tumor metastasis and therapy process in a clinical setting.** The TAMs, including CPs, ctDNA, CTCs, EVs, would be released into the circulation and can be employed to detect minimal tumor generation and monitor tumor heterogeneity. When the tumor is formed, CTCs will be generated by the primary tumor and invade the circulation. Original CTCs mostly cooperate with TEPs to enhance their viability and enter circulation as single cells or CTC clusters; otherwise, they form apoptotic bodies via immune phagocytosis. EVs then act as pre-metastatic scavengers that can resist immune damage and facilitate metastasis in secondary tumor areas. After progressive application of targeted therapeutic measures, those drug-resistant cancer cells will dramatically proliferate by adaptive evolution. In an example of colorectal cancer with EGFR targeted therapy, the mutations such as KRAS^G12D^, KRAS^Q61H^, and EGFR^G465R^ can be detected via ctDNA during regular monitoring 85, during which the formation of pre-metastasis or relapse could be predicted and managed so as to avoid tumor cell dissemination. Natural cancer development is indicated by the red arrows, whereas the development of tumors after therapy is shown by the black arrows.

Surviving cancer cells tend to develop drug resistance due to mutation and evolutionary selection when different therapeutic means are employed on heterogenic tissues (Figure 3) 33. Therefore, deciphering the heterogeneity of tumors may help clarify the mechanism of drug resistance and enhance the performance of individualized drug therapy 34, 35. Recent studies have revealed that recurrent estrogen receptor 1 mutations may play a critical role in acquired endocrine therapy resistance 36. Despite the fact that both *N-ras* and *phosphatidylinositol 3-kinase* mutations in ctDNA can predict drug resistance against monoclonal antibodies targeting EGFR, potential negative errors have been observed due to biological heterogeneity 37.

Integrated liquid biopsy might be an effective means of eliminating such discrepancies and probing unknown mutations. RNA-seq is a typical form of detection included in elementary integration, and a recent ctDNA profiling method known as cancer personal profiling by deep sequencing (CAPP-seq) found a high frequency of inter- and intrapatient heterogeneity in resistance mechanisms after initial EGFR tyrosine kinase inhibitor therapy 38. Additionally, intermediate integration of ctDNA and exoRNA/DNA, combining two different biological processes (EVs are shed by living cells, while ctDNA is shed by necrotic/apoptotic cells), would facilitate longitudinal surveillance of drug resistance. Additionally, ctDNA-exoRNA integration was reported to increase the sensitivity of *EGFR* mutation detection in plasma, and significant improvement was observed in NSCLC patients without distant metastasis 32. ctDNA-exoRNA integration yielded 92% sensitivity and 89% specificity for *EGFR* T790M detection, overcoming the limitation of low T790M abundance in the blood (58% sensitivity and 80% specificity using an FDA-approved cobas® test) 39 40. Meanwhile, EV-ctDNA integration may elicit compensated information because resistant cell-originated EVs reveal alterations in the cancer microenvironment, and sensitive cell-originated ctDNA may indicate mutation evolution. Furthermore, a continuous individualized risk index (CIRI) method has been introduced as a form of advanced integration for cancer therapy 14. The CIRI estimates an individual's outcome at any given time point through a Bayesian methodology based on 6 parameters, including three established risk factors: the International Prognostic Index (IPI), the molecular features of the cell of origin, and interim imaging, as well as three ctDNA risk factors (pretreatment ctDNA levels, early molecular response, and major molecular response). CIRI has been shown to be useful as a predictive biomarker for therapy selection. Therefore, the strategy of risk profiling can potentially increase the precision of personalized therapy selection.

## Mechanistic insight into integrated liquid biopsy for improved accuracy

Dozens of studies employing integrated liquid biopsy have demonstrated its effectiveness in improving the sensitivity of cancer diagnosis, suggesting that this strategy is ideal for cancer management. Therefore, it is highly desirable to elucidate the biological origins and mutual interactions of different biomarkers in anticipation of clinical applications.

CPs are secreted from diverse human cells, including immune cells and tumor-associated cells, and were the first such biomarkers to be exploited and commercialized. The demonstrated high specificity of CPs in reflecting tumor-associated characteristics makes them attractive for cancer surveillance. In contrast to CPs, ctDNA fragments are principally released from apoptotic or necrotic cells. Given the short circulating time of ctDNA (ranging from 16 min to 2.5 h), the dynamic and continuous monitoring of ctDNA fragments plays a paramount role in cancer prediction 41. However, the tendency of ctDNA to degrade rapidly makes its accurate detection particularly challenging. In order to improve the detection efficiency of free serum protein and nucleic acid, several strategies have been applied. Conventionally, an enrichment strategy was employed to purify ctDNA and RNA prior to further sequencing because of their low concentrations in the complex liquid and their susceptibility to interference from complicated substrates 42. Meanwhile, tumor-specific methylation is abundant in the early stage of cancers, when the release of ctDNA into the blood is lowest 43, 44. Therefore, the combined detection of ctDNA and methylation simultaneous would increase the sensitivity and accuracy in the early detection of cancer.

EVs, comprising exosomes, microvesicles, and apoptotic bodies, are free-floating bodies enwrapped by lipid rafts and contain tumor-specific signatures of nucleic acids and proteins. Briefly, exosomes emerge by budding in multivesicular bodies and are released into the plasma by fusion of multivesicular bodies with the cell membrane, whereas microvesicles and apoptotic bodies are leaked from dying cells due to external stimulation. Notably, selective accumulation of certain functional mRNA, microRNA, and protein species in microvesicles can occur. In contrast to normal cell sprouting, CTCs are spontaneously released from primary tumors to the peripheral blood circulation, constituting seeds for subsequent metastases in distant organs 45. CTCs also have a short half-life of 1 to 2.4 h in blood vessels 46, which may provide dynamic disease information regarding ongoing metastasis and the progression of disease status 47. Because trace CTCs and EVs cannot be amplified directly *in vitro,* it is necessary to use an indirect strategy involving an enrichment step prior to detection e 48.

Integrating methods with distinctive principles to strengthen integrated liquid biopsy is likely the most reliable strategy for ideal cancer management. The quantification and characterization of CTCs/EVs represent major technological challenges in liquid biopsy due to the extremely low concentrations of these targets. All the methods developed to quantify CTCs and EVs in the peripheral blood could be categorized as biological or physical. Biological methods are typically based on antigen-antibody or ligand binding; these methods are involved in typical tests for *epithelial cell adhesion molecule (EpCAM)*, human EGFR2, PSA and oncolytic viruses 49. Physical methods are mainly filter-based processes that can capture CTCs according to size differentiation or utilize ultracentrifugation to separate EVs 50. *In vivo* CTC enrichment using either an inserted metal wire or magnetic separation 51 and the *in vitro* integration of biomimicry and nanotechnology have remarkably improved capture efficiency 52. Once CTCs are enriched, patient-derived CTCs can be characterized by single-cell proteomics through microfluidic western blots as well as integrated trapping and encapsulation of single cells, offering a complementary taxonomy for understanding CTC biology and translating it to clinical use 46, 53, 54. Despite the fact that methods for EV enrichment are identical to those applied for CTC separation, specific approaches have especially been exploited for size-based exosome isolation, such as asymmetric flow field-flow fractionation (AF4) and centrifugation 55, 56.

Tumorigenesis, metastasis and tumor evolution are the typical forms of cancer development, and the above-noted biomarkers can reflect the accumulation of either genetic mutations or epigenetic modifications (Figure 3). During early tumorigenesis, ctDNA can be used to assess diseases from just a few million malignant cells before radioscopy is feasible 57. Additionally, both exosomes and ctDNA can be released from all composites of cancer niches to trigger tumor growth and metastasis, suggesting their potential in very early-stage detection and predicting tumor growth and metastasis 58, 59. As a tumor grows, cancer cells, as either single CTCs or CTC clusters, tend to either break through the extracellular matrix and infiltrate the circulation or transform into EpCAM^+^ CTCs, which reduces cell adhesion and promotes polarization and metastasis. Meanwhile, platelets immediately adhere and mechanically protect CTCs from anoikis or destruction by forming a cell fibrin-platelet aggregate surrounding CTCs 60. These processes allow TEPs to express abundant cancer-specific RNA to inflict shear stress, oxidative stress and immune system responses, which can be leveraged as biomarkers in cancer detection 61. Therefore, CTC counts of >5/7.5 mL of whole blood are the gold standard to evaluate metastasis and relapse 62. Furthermore, CTCs and CTC clusters with high metastatic potential usually feature hypomethylation of stemness- and proliferation-associated genes, which usually leads to poor prognosis and tumor metastasis^63^. In addition, the source of a tumor can be absolutely confirmed through integrated detection of various tumor-CpG methylation patterns 64, 65. EVs containing cancer-related factors can also contribute to premetastatic niche initiation, malignant conversion and immune resistance 66. As a result, EVs are being intensively investigated for their ability to indicate tumor heterogeneity and their probable participation in homeostatic maintenance of the tumor microenvironment 67 as well as decreased angiogenesis 68, metastasis 69, and drug resistance 70 associated with the initiation of a premetastatic niche.

In summary, any detection approach based on a single biomarker reflects limited information that may result in misleading predictions regarding tumor progression. Thus, integrating multiple biomarker approaches provides comprehensive information that may compensate for the drawbacks of single biomarkers in facilitating early cancer detection and intervention (Table 1). Meanwhile, integrated methods capable of dynamically tracking biomolecule alterations are highly desired for accurate and reliable diagnosis. A direct detection approach is most suitable for high-concentration CPs and amplifiable nucleic acids. In contrast, an indirect strategy involving an enrichment step followed by a detection step is specifically suitable for trace CTCs/EVs that cannot be amplified directly *in vitro* 48. Technological innovations have enabled reliable CTC-EV-ctDNA integration from various molecular species containing protein, RNA, and DNA (Figure 2), providing further clinical insight beyond the information provided by *in silico* target prediction.

## Cost-effectiveness of integrated detection in clinical practice

Early and accurate cancer diagnosis is the key to reducing the economic burden of cancer. Classical tissue biopsy has arguably presented a heavy burden according to US Medicare analysis: as of 2017, the average cost of a solid biopsy for lung cancer is $8,869, and the total cost per patient will reach $37,745 for the 20% of needle biopsies that lead to follow-up complications. Compared with single liquid biopsy alone, integrated liquid biopsy provides improved cost-effectiveness in the following ways. First, integrated liquid biopsy is less expensive per marker than its counterpart. For instance, the current cost for single ctDNA mutant detection with digital PCR is approximately $300, while the cost for elementarily integrated detection of all ctDNA mutants with DNA sequencing is < $1,000 with Illumina 71. Second, combining various biomarkers in an all-in-one format is superior to any single-biomarker method alone in terms of noticeably improved sensitivity, decreased turnaround time, and a relatively reasonable cost (Table 1). For example, researchers from Johns Hopkins University developed an extremely inexpensive multianalyte blood assay (less than $500) with excellent specificity and sensitivity for cancer diagnosis 7, 32. Third, integrated liquid biopsy allows dynamic tracking of cancer development to assist in drug selection; in this manner, the cost of nontargeted tumor medication can be avoided. For instance, ctDNA profiling demonstrated that *KRAS* mutant cells effectively drive cancer progression after the suppression of the *MEK1* mutant population by panitumumab and trametinib 72. Recent multicenter clinical trials on IsoPSA assays revealed that over 40% of biopsies could be avoided by employing the unique ratiometric parameter K. This parameter reflects the entire spectrum of structural changes to PSA and may thus lower the likelihood of overdetection and the overtreatment of nonlethal PCs (44).

## Future strategies for effective integration

Several fundamental questions need to be answered prior to effective integration under physiological and pathophysiological conditions: What is the relationship between the degree of ctDNA variations and the RNA/protein abundance of hundreds of functionally coherent gene sets? What are the differences between primary circulating biomarkers such as ctRNA or CP and secondary biomarkers such as RNA/protein analysis from EVs or CTCs? Will different cancer stages (epithelial-to-mesenchymal transition (EMT) and mesenchymal-to-epithelial transition (MET)) or tumor cell status (primary or circulating) significantly affect the spatial and temporal distributions of ctDNA or exoRNA? Understanding the gene regulatory networks of biomarker integration will lead to significant insights and has tremendous implications for cancer intervention and management. Therefore, for integration detection, the core purpose is to reflect the real developmental stage and increase therapy efficiency for cancer.

How can users predict and confront the inconsistent results stemming from different biomarkers or detection methods in integrated detection? Considering the complexity of tumorigenesis, any single-marker-based diagnosis will have a risk of error, integrated detection may naturally increase the possibility of exposing these errors by presenting contradictory results, by which the error can be significantly reduced in further diagnosis.

What types of integration could be optimal? Both elementary integration and intermediate integration can provide characteristic molecular profiles of cancers in the spatial scale. Advanced integration might provide integrated information in a higher dimension by combining these comprehensive evidences, including medical imaging, tissue diagnosis, and molecular profiling, to map the characteristics of a cancer in detail based on the complementarity of these results.

How can we establish a risk prediction model using the acquired integrated data from a time series? Integration in the temporal domain will help us characterize the law of cancer progress or metastases. Therefore, combining the dynamic changes in various biomarkers with previous medical records would facilitate the prognosis analysis of cancers. It is highly plausible that this type of dynamic method focusing on the whole process, including patient diagnosis and treatment, will become the mainstream of prognostic analysis in the near future.

**Figure 4:**
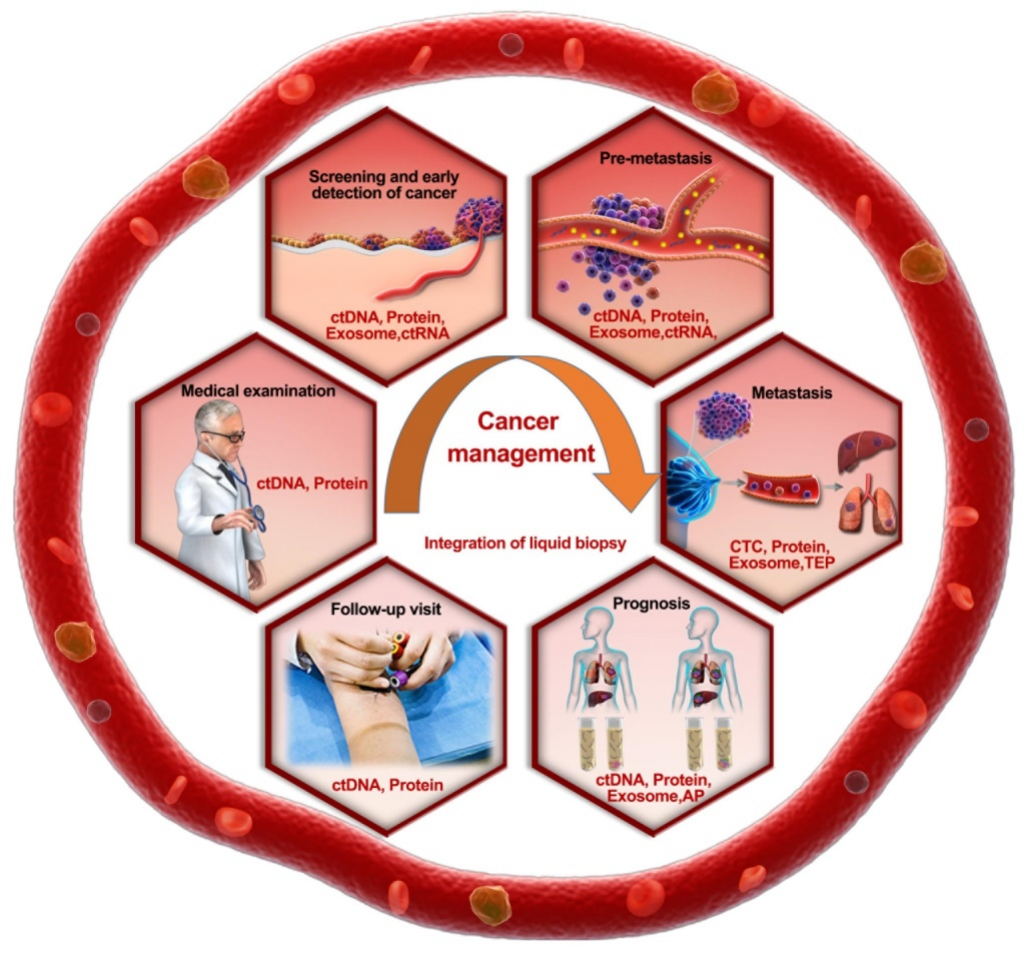
** Roadmap of the rational selection of integrated TAMs during liquid biopsy in cancer prevention and management.** Recommended biomarker combinations are proposed for different stages of cancer management.

The feasibility of integrated liquid biopsy is largely guaranteed by the quality control and standardization of each detection method. In particular, the standardization of annotations resulting from liquid biopsy is challenging due to varied cohort compositions (sex, age, and cancer stage) and diverse environmental factors (sample collection, storage, and detection methods) when analyzing the same biomarker. For instance, noticeable discordance exists among different populations: the *KRAS* mutation rate reached 94% in a pancreatic intraepithelial neoplasm population 73, whereas it was only 30% in a population involving 221 patients with surgically resectable pancreatic cancer patients, and ctDNA-CP integration merely increased the sensitivity to 64% 8. Thus, choosing an optimal biomarker panel and a self-controlled design that can feasibly eliminate errors in distinctive populations is recommended (Figure 4).

Integrated liquid biopsy produces data that are substantially more complicated than data from any single biomarker, requiring a canonical big data processing methodology. Deep learning has recently been introduced to assist with diagnostic and therapeutic decision making by reading patients' medical images 74, 75. AI-enabled selection of gene panels has been developed to diagnose cancer from TEPs 61, and machine learning was integrated in CancerSEEK to localize the primary source of cancer, which largely solved the notorious inefficiency in determining cancer types 7. Empowered by various mathematical models, integrated liquid biopsy is anticipated to give improved answers to the following questions (Figure 1): what biomarker combination represents the at-risk population at each cancer stage, and what are the optimal algorithms for interpreting positive findings from inconsistent methods? Despite promising prospects for assisting diagnosis, AI also has its own limitations due to its early stage of development: inadequate sample size or unrepresentative sample characteristics may lead to model misspecification or errors in decision making, as the precision of AI largely depends on the sample size for deep learning, the dimensionality character numbers of the sample, and the algorithm itself 76. Furthermore, choosing the optimal algorithm is critical; for example, a “win-probability” model could provide a simple method to overcome the difficulty encountered by a machine learning approach in interrogating results across patient datasets 77.

## Conclusions

Liquid biopsy has demonstrated unparalleled advantages over conventional tissue biopsy and medical images in terms of earlier cancer detection and better surveillance of cancer metastasis and prognosis. Different from conventional imaging approaches, which mainly reveal changes in tumor size, profiling the dynamic distribution of various tumor-associated molecular biomarkers may provide continuous genetic mutation information, which will reflect different cancer stages and provide improved guidance for clinical therapy. Furthermore, the rational integration of liquid biopsy, solid biopsy, and medical imaging would prevent discordance or disagreements caused by tumor heterogeneity or individualized analytical methodologies. This advantage would facilitate cancer prevention and early intervention.

Integrated liquid biopsy covers both the integration of different detection methods and the combination of multiple biomarkers, including proteomics, genomic sequencing, and DNA methylation profiling, that could reveal mechanistic differences in the development of different cancers. During carcinogenesis, integrated biomarkers could provide an earlier diagnosis than any single biomarker alone. Furthermore, ctDNA could identify the position of the tumor by detecting DNA methylation in an accurate and noninvasive manner 43, 44.

Socioeconomic factors should also be considered in the clinical implementation of the integrated liquid biopsy strategy. In the early stage, additional blood tests for enriching the database will carry relatively high costs. However, with the gradual growth of the database, we will benefit from the mathematical model as it provides increasingly accurate and specific guidance on cancer management.

## Figures and Tables

**Table 1:** Comparisons of the advantages and drawbacks of integrated and single liquid biopsy biomarkers.

Clinical stages	Features and Drawbacks of Single TAM			Categories and Advantages of Integrated TAM
		Features	Shortcomings	
**Early diagnosis**	**ctDNA**	Can detect a minimum of 0.005% abundance of mutant alleles. Its concentration correlates to tumor size and stage 17.	Limited sensitivity in the detection of non-necrotic cancers 78.	Elementary integration: Structure-based IsoPSA^TM^ assay based on PSA measurement, provides a net benefit against other protocols 26. Meanwhile combination of ten exosomal miRNAs yielded 0.99 sensitivity and 1.00 specificity in detection of early-stage ovarian cancer 9. Intermediate integration: exoRNA-ctDNA integration in the plasma of NSCLC patients improved the sensitivity of EGFR mutation detection from 26% to 74% 37. Advanced integration: CancerSEEK combines ctDNA and CP with machine learning and has achieved 98% sensitivity and a <1% false positive rate for the detection of five cancer types 7.
	**CP**	Convenient detection	Less informative about tumor mutations^79^.	
	**EVs**	Exosomes containing uncontaminated DNA, RNA and proteins provide outstanding specificity and sensitivity 21.	Lack of spatial and temporal tumor heterogeneity.	
	**CTCs**	A high concentration (≥5 CTCs/7.5 mL) indicates higher risk of early cancer progression.	CTCs in the blood are rare.	
**Therapy management (Postope-ration and prognosis**	**ctDNA**	Short circulating time contributes to the monitoring of tumor evolution in real-time 80.	Less efficient in analyzing drug-resistance mechanisms.	Elementary integration: DNA sequencing mutations in exosomes is reliable in monitoring pancreatic cancer to establish curative surgical therapy (41). Intermediate integration: exoRNA-ctDNA integration increases the sensitivity of *EGFR* mutations detection in plasma to monitor responses to therapy 37. CEA-ctDNA integration predicts recurrence after adjuvant chemotherapy 81. Advanced integration: Combination of liquid biopsy (biological information from CTCs) and MRI (anatomy and physiological information) provides additional information than either modality alone 82.
	**CTCs**	Key checkpoints in metastasis. Strong prognostic factor for overall survival in patients. Assist in the establishment of a CTC-derived xenograft (CDX) model to search for druggable targets 83.	Inefficiency in discovering intratumoral heterogeneity Inefficiency clonal evolution of cancers after targeted therapy.	
	**EVs**	Exosomes miRNA profiles can predict survival after therapy 84 .	Therapy-induced tumor heterogeneity and EV diversity.	

## Abbreviations

CPs: circulating proteins
ctDNA: circulating tumor DNA
ctRNA: circulating tumor RNA
EVs: extracellular vesicles
CTCs: circulating tumor cells
TEPs: tumor-educated blood platelets
PC: prostate cancer
CEA: carcinoembryonic antigen
PSA: prostate specific antigen
exoRNA: exosomal RNA
NSCLC: non-small cell lung cancer
PSA: prostate specific antigen
KRAS: kirsten rat sarcoma viral oncogene
EGFR: epidermal growth factor receptor
ABs: apoptotic bodies
AI: Artificial Intelligence
